# Effects of i‐PRF, A‐PRF+, and EMD on Osteogenic Potential of Osteoblasts on Titanium

**DOI:** 10.1111/cid.13406

**Published:** 2024-10-22

**Authors:** Liza Lima Ramenzoni, Jothi Varghese, Patrick Roger Schmidlin, Shubhankar Mehrotra

**Affiliations:** ^1^ Clinic of Conservative and Preventive Dentistry, Division of Periodontology and Peri‐Implant Diseases, Center of Dental Medicine University of Zurich Zurich Switzerland; ^2^ Department of Periodontology, Manipal College of Dental Sciences, Manipal Manipal Academy of Higher Education Manipal India

**Keywords:** enamel matrix derivative, injectable‐platelet‐rich fibrin, osteoblast, osteogenesis, platelet‐rich fibrin, titanium dental implant

## Abstract

**Objective:**

The study evaluates three biologically active substances with known bone‐inductive potential on previously decontaminated titanium (Ti) discs.

**Material and Methods:**

Rough and smooth Ti surfaces were contaminated with a multispecies biofilm and cleaned with a chitosan brush. Discs were treated either with injectable‐platelet‐rich fibrin (i‐PRF), advanced platelet‐rich fibrin (A‐PRF+), or enamel matrix derivatives (EMDs) before osteoblast seeding.

**Results:**

Biocompatibility, adhesion, migration, and gene expression of *runt‐related transcription factor 2* (*RUNX2*), *collagen Type I Alpha 2* (*COL1a2*), *alkaline phosphatase* (*ALP*), *osteocalcin* (*OC*), and *osteonectin* (*ON*) were performed. All the tested biologic agents similarly increased cell viability. Specifically, osteoblasts seeded over i‐PRF and EMD‐treated surfaces showed improvement in adhesion and migration and significantly increased *ALP, OC, ON, RUNX‐2*, and *COL1a2* mRNA levels up to 2.8 fold (*p* < 0.05) with no differences between Ti surfaces.

**Conclusions:**

i‐PRF and EMD possess beneficial bioactive properties that enhance tissue healing and promote regeneration on thoroughly sterilized surfaces. Biologically active materials may hold the potential to influence the process of implant re‐osseointegration, which warrants more research since sterilization of the affected surfaces under clinical conditions is still not reliably possible and remains one of the greatest challenges.

## 1 Introduction

Platelet‐rich plasma (PRP) was initially harnessed as a regenerative agent derived from one's own blood, by increasing platelet concentration through centrifugation. Its utilization has seen a substantial rise across multiple medical disciplines, including oral‐maxillofacial surgery [1]. PRP is renowned for its ability to facilitate bone and tissue healing, owing to the increased levels of blood‐derived growth factors that influence cellular growth, morphogenesis, and differentiation [2, 3, 4, 5, 6, 7, 8]. In the dental field, numerous studies underscore the beneficial impact of PRP in various applications, including guided bone regeneration [9], early bone formation around implants [10], its combination with bone grafts for maxillary sinus floor augmentation procedures [11], and the regeneration of periodontal intrabony or furcation defects [12]. Despite its burgeoning popularity, concerns have arisen regarding the concurrent use of anticoagulants, such as bovine‐derived thrombin, which has been shown to hinder wound healing by impeding clot formation [13], a crucial step in tissue healing. In response to these concerns, Platelet‐rich fibrin (PRF) was developed as a second‐generation autologous platelet concentrate, devoid of anticoagulants or other additives [14]. Recent systematic reviews have documented the long‐term effects of PRF on tissue wound healing [15, 16, 17]. PRF offers two notable advantages: it contains host immune defense cells (leukocytes) that combat infection [18], and in its solid form of a fibrin clot it can serve as a three‐dimensional scaffold, expediting the healing of bone and gingival tissues by continuously releasing growth factors [14, 19]. Various PRF preparation protocols are currently in use, including A‐PRF+, formulated with low relative centrifugation force (RCF) to enhance platelet and leukocyte content, thereby promoting the release of growth factors and the proliferation of fibroblast cells [20]. Lower RCF and the use of specific tubes yield i‐PRF, a liquid form of PRF containing platelets, white blood cells and fibrinogen, that can be employed either on its own or in conjunction with other biomaterials to stimulate cellular functions via release of growth factors for tissue repair [21, 22]. Alternatively, enamel matrix derivative (EMD) supports the healing of connective tissues, stimulating various mesenchymal cell types, and enhancing the expression of tissue‐specific markers such as alkaline phosphatase, collagen, and osteocalcin within osseous tissues, improving implant osseointegration. These biological effects render EMD a valuable tool in periodontal regenerative procedures and in peri‐implantitis therapies [23].

Dental Ti implants have been identified as an ultimate therapeutic modality for restoration of missing teeth owing to their predictable success rate. Lately, researchers are exploring strategic modifications on the implant surface such as roughened topography and addition of bioactive materials to enhance osseointegration into the surrounding tissues [24, 25]. Ti implant surface roughness influences cytokines, growth factors, and osteoblastic cell activities [26]. Additional application of biologically active materials possess optimal soft tissue healing and osteopromotive properties which could warrant its use for the management of peri‐implant diseases [27, 28]. Literature search demonstrates a substantial increase in survival rates of osteoblasts and other cellular events, with use of i‐PRF which warrants its positive role in bone tissue formation [21, 29].

In a recent study, the addition of PRF to alveolar sockets receiving titanium implants demonstrated a significant enhancement of implant stability in the early healing period [30]. Also, when titanium discs were immersed in liquid platelet concentrate for 10 min, they demonstrated a significantly thicker surface fibrin network formation on the surface as compared to nontreated ones [31]. It was hypothesized that this biologic coating may contribute to enhanced cell migration and differentiation on the surface of the implant. Also, it has been demonstrated that i‐PRF induced significantly higher cell migration, differentiation, and release of growth factors, such as platelet‐derived growth factor (PDGF) and transforming growth factor (TGF) on fibroblasts [32]. These studies reinforce the positive biologic influence; i‐PRF coating can play, on the decisive biologic events taking place in the immediate vicinity of implant after its placement. Nevertheless, the complete understanding of the role and significance of PRF on its osteogenic potential is still lacking.

Accordingly, the objective of the present in vitro study was to provide new comparable insights into osteoblasts cell response on decontaminated smooth and rough Ti discs coated with either i‐PRF, A‐PRF+, or EMD. We hypothesized that the conditioning of Ti discs (both smooth and rough) with any of the experimental bioactive agents may not induce significant difference in osteoblastic activity which predominantly contributes to periodontal soft tissue healing and osseous regeneration in implant therapy.

## 2 Materials and Methods

### 2.1 Development of Biofilm Onto Ti Discs, Cleaning, and Decontamination

**FIGURE 1:**
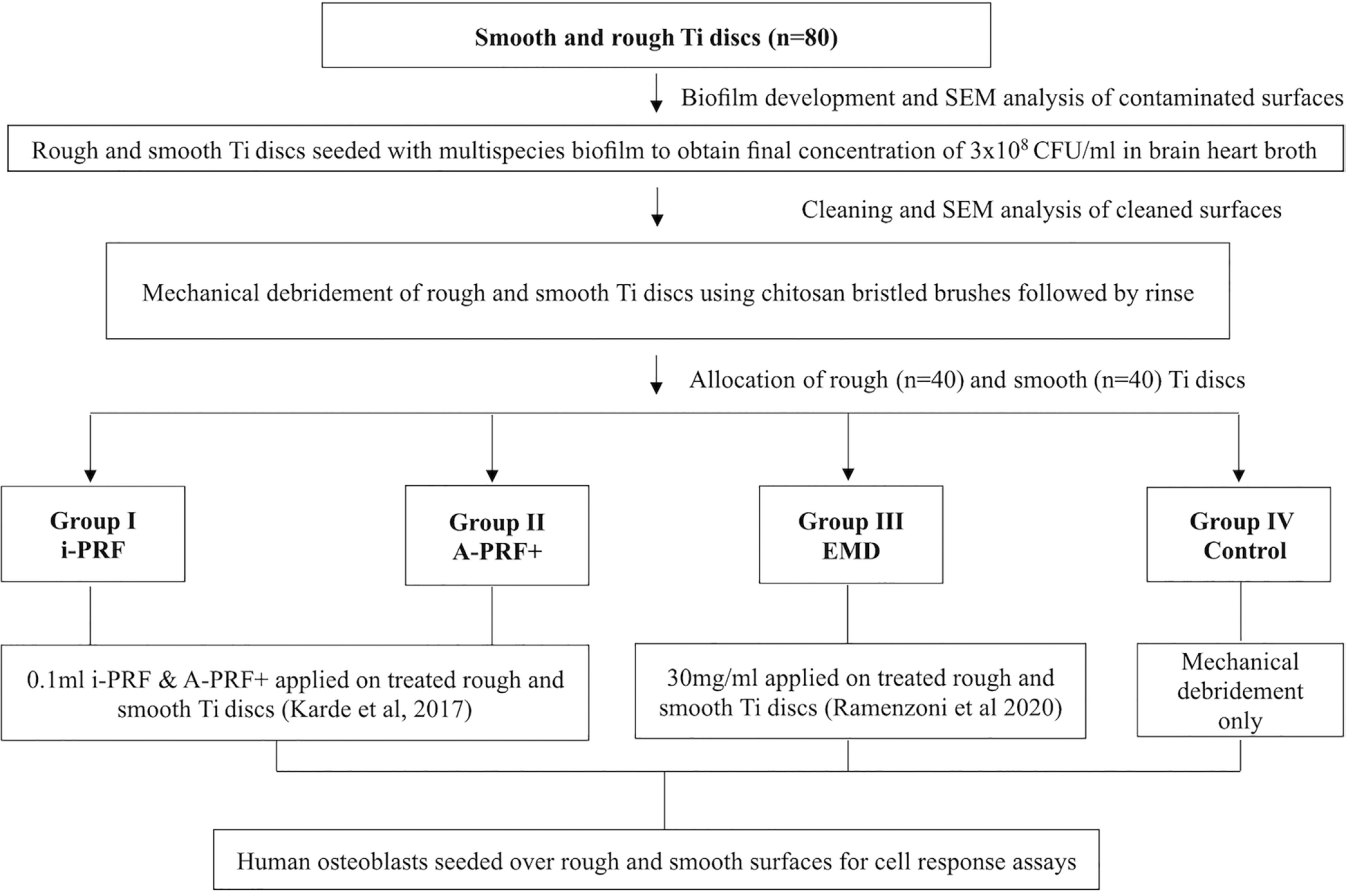
Methodology for development of biofilm on Ti discs followed by cleaning and further osteoblast response assessment.

**FIGURE 2:**
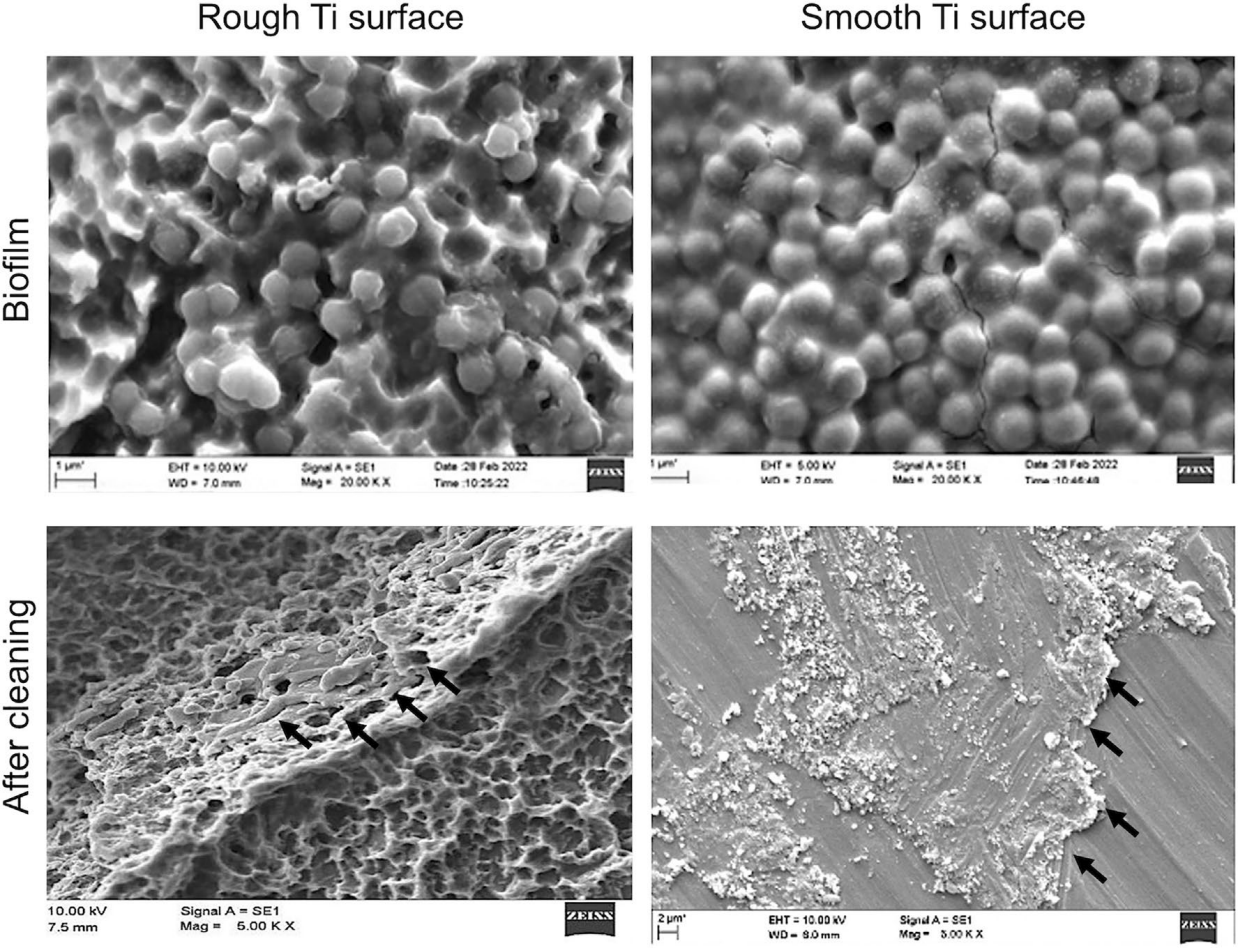
Representative SEM images of the two different titanium surfaces (smooth and rough) with biofilm followed by application of decontamination method with chitosan bristled brush. The untreated titanium surfaces are covered by a dense bacterial biofilm. After cleaning and decontamination, biofilm residues are still visible in SEM images on the microstructure titanium surfaces (arrows). Magnification SEM: with biofilm: ×20 000; after cleaning: ×5000.

The methodology for development of biofilm on Ti discs followed by decontamination, cleaning, and cell culture assessment is described in the flow diagram (Figure 1). Smooth Ti discs and sandblasted and acid‐etched rough Ti (Ti6Al4V) discs, 15 mm in diameter/1‐mm thickness were kindly provided by Straumann AG (Institute Straumann AG, Basel, Switzerland). All the discs were seeded with multispecies biofilm consisting of *Porphyromonas gingivalis* ATCC 33277, *Prevotella intermedia* ATCC 15033, *Tannerella forsythia* ATCC 4304, *Treponema denticola* ATCC 35405, *Actinomyces oris* 27044, *Streptococcus anginosus* 3397, and *Streptococcus oralis* 10557. In brief, bovine dermal type I collagen (10 μg/mL) collagen in 0.012 M HCl in water (Gibco, Fisher Scientific, MA, USA) was applied on the Ti discs and stored overnight at 4°C. This step was performed to replicate the elimination of inherent bone constituents which take place in peri‐implantitis as a result of osteoclastic resorption. The precoated Ti discs were placed into a 24‐well tissue culture plates containing 2 mL of bacterial suspension and incubated in the anaerobic environment at 37°C for 21 days with renewal of the suspension every week. All the bacterial strains were cultured to produce a final concentration of approximately 3.0 × 10^8^ CFU/mL in brain heart broth (Sigma‐Aldrich, Merck KGaA, Darmstadt, Germany). For the cleaning, smooth and rough TI discs were debrided using chitosan bristled brush inserted into an oscillating handpiece (average speed 700 rpm, Labrida Bioclean, Institut Straumann AG, Peter Merian Basel, Switzerland). Cleaning effects were screened by morphologic analysis of the contaminated, and decontaminated surfaces of discs were conducted using a scanning electron microscope (JSM6010, JEOL, Tokyo, Japan) to analyze the changes occurring on the disc surfaces. A standard working distance of 5–10 mm using magnifications from 5000× to 20,000× was utilized. To confirm the development of mature polymicrobial biofilm, one Ti disc (both smooth‐ and rough‐treated) were subjected to SEM analysis and representative images were taken (Figure 2). Furthermore, SEM images from discs from each group (smooth and rough) were analyzed after decontamination with chitosan bristled brush. SEM examination revealed the decontamination protocol was effective in elimination of biofilm and did not display any perceptible alterations on the Ti discs.

### 2.2 Preparation of i‐PRF and A‐PRF+ and Application of Biological Agents on Decontaminated Ti Discs

For preparation of PRF variants, venous blood samples were collected antecubital from six healthy volunteers (ASA I) aged 25 to 35 years. The institutional review board approved the study (IEC1:24/2022) in accordance with the Declaration of Helsinki. An informed consent was obtained from the volunteers prior to the blood donation process. The PRF formulation process was as per the previous protocol [15, 22]. For A‐PRF+, two sterile glass tubes with 9 mL of whole blood were centrifuged in a fixed‐angle centrifuge with a radius of 110 mm (DuoQuattro, Avtec surgical, Mount Pleasant, SC, USA) following manufacturer's instructions at 1300 rpm/208 g × 8 min. After the designated centrifugation steps, the tubes were kept vertical for 5 min [33]. The fibrin clots located in the middle part of the tube, between the red corpuscular phase at the bottom of the tube and the acellular plasma at the top were harvested for the experiments using a forceps and sterile gauzes. They were further mechanically compressed to A‐PRF matrices in an expression kit (Intralock, Boca Raton, FL, USA). For the i‐PRF preparation, two plastic tubes with 9 mL of whole blood was transferred into a vacutainer devoid of anticoagulant and immediately centrifuged at 700 rpm for 3 min resulting in RCF of 60g [22]. 1 mL from the upper liquid fibrinogen layer above the buffy coat (BC) was manually aspired using blunt needles and a micropipette without including any erythrocytes. Furthermore, the i‐PRF samples were mixed in plastic culture dishes with 5 mL of Dulbecco's modified eagle medium (DMEM, Sigma‐Aldrich Chemie GmbH Taufkirchen, Germany) for further experiments [22]. Four experimental groups with 10 titanium rough Ti and 10 smooth Ti discs in each group (total *n* = 80) were treated as follows: (a) Group I: i‐PRF‐, (b) Group II: A‐PRF+, (c) Group III: EMD, and (d) Group IV: control discs (only mechanically debrided). For the biological agents application on the Ti discs in group I, 0.1 mL of i‐PRF was applied using a micropipette; For group II, parts of a cut A‐PRF+ matrix were separately applied on the surface of the treated Ti discs (both smooth and rough from both groups I and II). Furthermore, these Ti discs were inserted into the culture plate wells containing 2 mL antibiotic and serum free DMEM under aerobic conditions at 37°C for 48 h. For group III, 30 mg/mL of EMD (Emdogain, Straumann AG Basel, Switzerland) was used based on relevant literature documented in previous studies [34, 35]. And in Group IV, the discs were debrided mechanically without the use of biological agents.

### 2.3 Cell Culture

The primary human osteoblast cells were donated by the Department of Operative Dentistry and Periodontology, University of Freiburg, Germany. Approval to conduct cell study was granted by the Ethics Committee of the Albert‐Ludwigs‐University Freiburg for research involving humans (EK153‐15) and informed consent obtained from the donors, in accordance with the Declaration of Helsinki. Detailed information about cell isolation and phenotype characterization are described in the previous study [36]. Cell cultures were carried out with (DMEM, Invitrogen, Carlsbad, California) supplemented with 10% fetal bovine serum (FBS, Invitrogen, Carlsbad, California), 100 units penicillin, and 100 μg/mL Streptomycin (Biochrom, Berlin, Germany) in an incubator at 37°C, in a 5% CO_2_ atmosphere, at 95% humidity. Induction of osteogenesis was promoted by culturing the cells with 50 μg/mL ascorbate, 2 mM β‐glycerophosphate, and 10 nM dexamethasone. Osteoblasts cells were seeded (5 × 10^4^ cells/well) on cell culture plates and grown to 80% confluence. Subsequently, the cells were washed with phosphate‐buffered saline (PBS 1×, Seromond, Biochrom, Berlin, Germany) and resuspended with 0.25% trypsin to enable further passages for experiments. Cells were seeded onto smooth and rough Ti discs previously treated with i‐PRF, A‐PRF+, and EMD. Cells were used between passages 6 and 10, and medium was replaced twice weekly.

### 2.4 Cell Viability Assay

The influence of i‐PRF, A‐PRF+, and EMD application on cell viability over Ti surfaces was determined by nonradioactive, colorimetric MTT assay (3‐(4,5‐dimethylthiazol‐2‐yl)‐2,5‐diphenyl tetrazolium bromide colorimetric, Sigma‐Aldrich, Steinheim, Germany). The osteoblasts were seeded over rough Ti discs (1 × 10^5^ cells/well) on 24‐well plates and incubated for 24 h at 37°C. Then, the cells were separately treated for 24 h with different biomaterials (i‐PRF, A‐PRF+, and EMD). Next, MTT (0.45 mg/mL) was added, and the cells were incubated for further 4 h at 37°C. Finally, after removal of MTT, dimethyl sulphoxide as a solubilization reagent was added, and the cells were additionally incubated for 2 h. The test absorbance at 570 nm and reference absorbance at 630 nm were measured using a spectrophotometer reader (Tecan, Austria). In total, 60 discs were used for three independent experiments replicated at least in three times.

### 2.5 Cell Adhesion, Proliferation, and Cell Migration Assays

Osteoblasts were seeded onto titanium discs in 24‐well plates at a density of 10 000 cells per Ti structure and cultured for 24, 48, and 72 h for the adhesion assay. For counting the cell number, 40,6‐diamidino‐2‐phenylindole (DAPI) was applied to visualize the nuclei as previously described. At each time point, the Ti structures were washed with PBS to remove nonattached cells and fixed in 4% formaldehyde for 10 min followed by staining with DAPI. Images were captured on an inverted microscope (DMi1, digital camera MC170, Leica, Wetzlar, Germany). Ten fields of view were captured per sample, and nuclei was counted using Image J software (Bethesda, MD, USA). The migration assay was performed using 24‐well plates and polyethylene terephthalate cell culture inserts with a pore size of 8 um (Costar, Corning Inc., Corning, NY, USA). The 20% platelet conditioned media in DMEM containing 10% FBS was filled into the lower compartment of the wells onto implant surfaces or controls. After starving the cells in DMEM containing 0.5% FBS for 12 h, 10 000 cells were resuspended and seeded in the upper compartment. After 24 h, cells were fixed with 4% formaldehyde for 15 min. Thereafter, cells were stained with 0.1% crystal violet solution (GoodBio Technology Co., Ltd., Wuhan, China) for 10 min. The upper side of the filter membrane was rinsed and gently wiped by a cotton swab to remove the cell debris. Images on the lower side of the filter were taken under an inverted microscope (DMi1, digital camera MC170, Leica, Wetzlar, Germany).

### 2.6 Gene Expression Analysis

Gene expression was investigated by real‐time quantitative polymerase chain reaction (RT‐PCR). Approximately, 50 000 osteoblasts were seeded onto smooth and rough Ti discs with EMD, A‐PRF+, and i‐PRF in 24‐well plates. After 7 days of culture, total RNA was isolated from cells using Total RNA Miniprep Kit (AXYGEN, Union City, CA, USA) according to the manufacturer's protocol. The RNA concentration was determined by a NanoDrop 2000 UV–Vis spectrophotometer as previously described. A total of 1‐μg RNA solution was immediately reverse transcribed to cDNA using a First Strand cDNA Synthesis Kit (GeneCopoeia, Rockville, MD, USA), and the final volume is 100 μL. Three independent experiments were performed for genes with the specific primers (Table 1). RT‐PCR was performed using 20 μL final reaction volume of RT‐PCR Mix Kit (GeneCopoeia, Rockville, MD, USA), and the target gene expression was assayed on an RT‐PCR Detection System. The ddCt method was used to calculate gene expression levels relative to house‐keeping gene *GAPDH* and normalized to control cells (blank well without Ti structure). Each sample contained pooled mRNA collected from three titanium surfaces, and all samples were log‐transformed. The experiments were performed in triplicate with three independent experiments.

**TABLE 1:** Primer sequence for reverse transcription‐quantitative polymerase chain reaction.

Gene	Forward primer (5′–3′)	Reverse primer (5′–3′)
*RUNX2*	GCC GGG AAT GAT GAG AAC TA	GAG GCA GAA GTC AGA GGT GG
*COL1a2*	GAG GGC AAC AGC AGG TTC ACT TA	TCA GCA CCA CCG ATG TCC AA
*ALP*	ACC ATT CCC ACG TCT TCA CAT TT	AGA CAT TCT CTC GTT CAC CGC C
*OC*	CAA AGG TGC AGC CTT TGT GTC	TCA CAG TCC GGA TTG AGC TCA
*ON*	AGC ACG GTA TTG TGA CTA ACT G	TCG AAC ATG ATC TGT GTC ATC
*GAPDH*	AAT CCC ATC ACC ATC TTC CA	TGG ACT CCA CGA CGT ACT CA

### 2.7 Statistical Analysis

The data compiled from the cellular activity were statistically analyzed using IBM SPSS software (IBM SPSS Statistics for Windows, version 23.0; IBM Corp., Armonk, New York). For the cellular activity of primary human osteoblast cells, the mean values and standard deviations were computed for the MTT test. Before conducting the analysis, the normality of the data was verified using the Shapiro–Wilk test, confirming that the data met the assumptions necessary for applying analysis of variance (ANOVA). Multiple comparison ANOVA with Bonferroni adjustment, with a global significance level of 5%, was then conducted to assess the statistical significance of the differences between the experimental groups. Differences were considered significant at *p* < 0.05. All experiments were performed in triplicate and repeated at least three times under the same conditions.

## 3 Results

### 3.1 Cell Viability, Adhesion, and Migration

**FIGURE 3:**
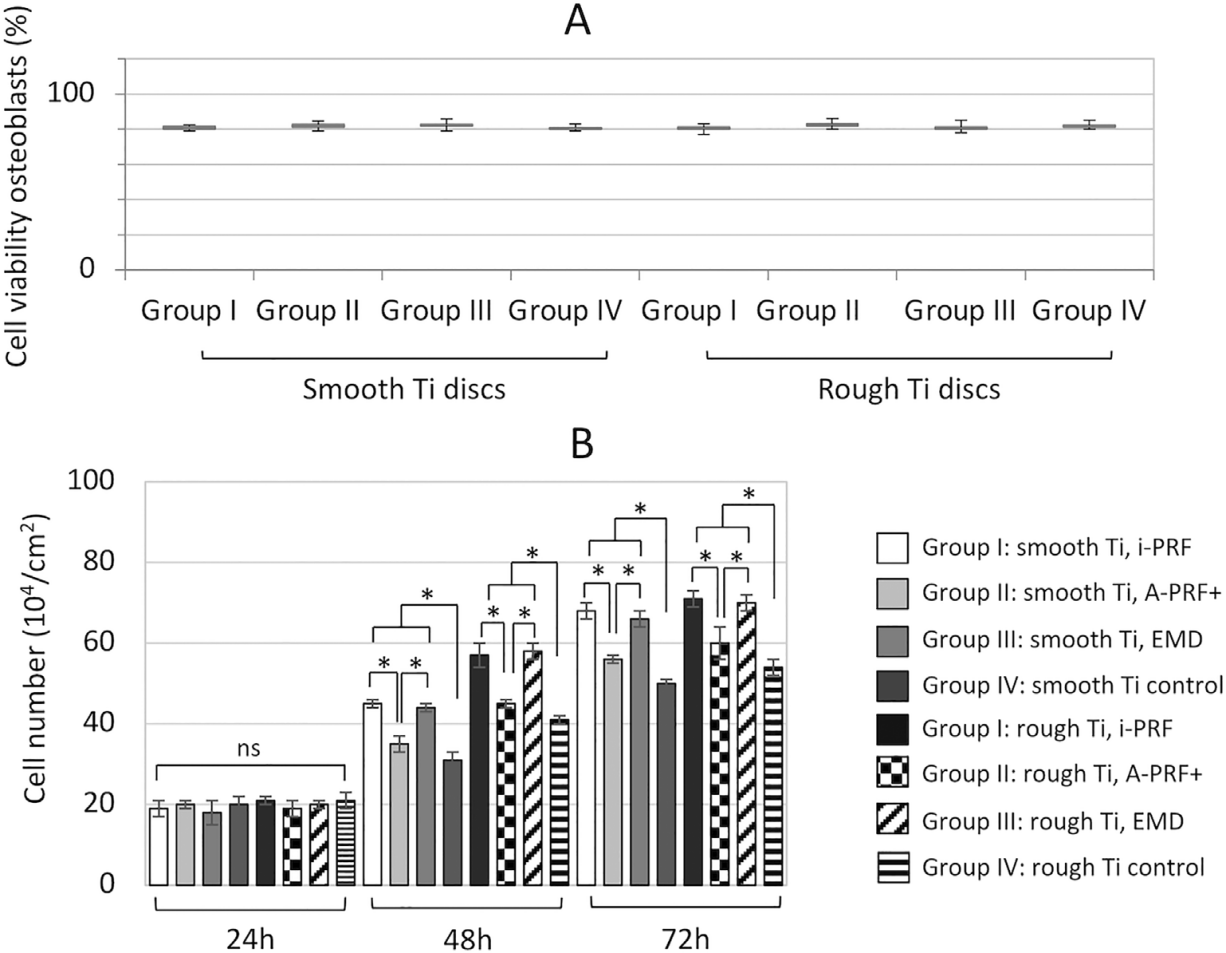
Cell viability (A) and cell adhesion (B) analysis of human primary osteoblasts quantified on smooth Ti (titanium) discs, sandblasted/acid‐etched/rough Ti discs followed by the treatment with injectable platelet‐rich fibrin (i‐PRF, group I), autologous platelet‐rich plasma (A‐PRP+, group II), and enamel matrix derivative (EMD, group III). Control group is smooth surface discs and rough discs without treatment (group IV). Cell adhesion was assessed at 24, 48, and 72 h. Scale bars = 100 μm, *A significant difference between control and experimental groups, *p* < 0.05.

**FIGURE 4:**
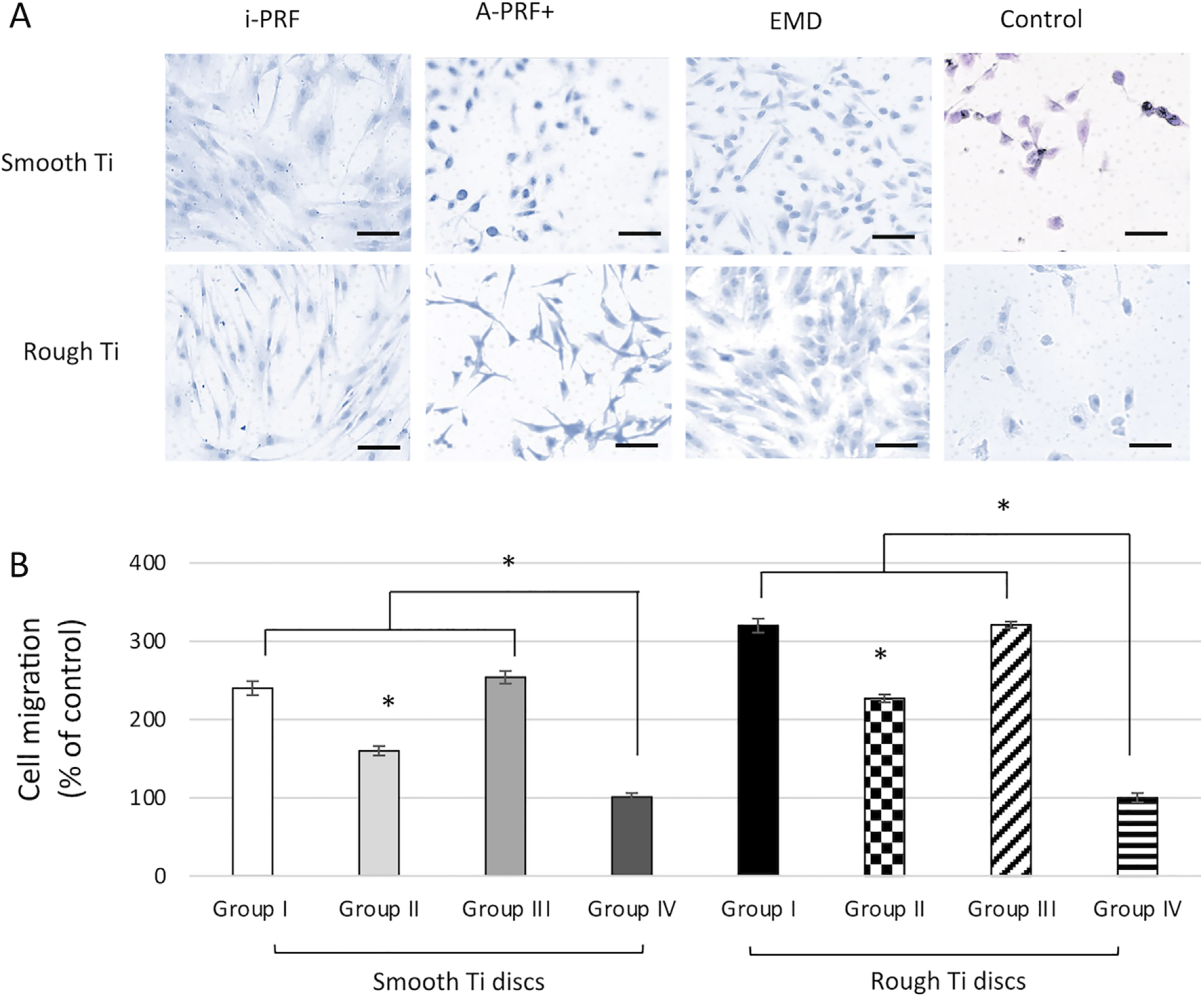
Effects of Ti surface topography (Ti smooth and Ti SLA) and i‐PRF (group I), A‐PRF+ (group II), and EMD (group III) treatment on the migration of human primary osteoblasts. (A) Cell migration was assessed after 48 h. (Scale bars = 100 μm). (B) Cell migration was quantified by normalizing to the control Ti smooth and SLA groups. *A significant difference between control and experimental groups, *p* < 0.05.

The findings of this study showed more than 75% cell survival on both smooth and rough Ti surfaces irrespective of PRF or EMD application. No significant difference in viability was detected in the intergroup comparison (*p* > 0.05) (Figure 3A). All the experimental biologic agents substantially improved cell viability and biocompatibility under the present in vitro culturing conditions (Figure 3A). Overall, all study groups seemed to have similar percentage counts, with higher values observed for group III (EMD) followed by A‐PRF+ and i‐PRF groups (*p* > 0.05, Figure 3B). Concerning the cell adhesion and migration assays, no significant difference between all the groups irrespective of the Ti disc surface topography or additional treatment (*p* > 0.05) was noticed at 24 h (Figure 4A,B). However, at 48 h and 72 h, the cells cultured specifically with i‐PRF (group I) and EMD (group III) showed significant improvement in osteoblast cell adhesion over the control (group IV, Figure 4A,B). Furthermore, cells cultured specifically on rough discs demonstrated slightly higher cell numbers in comparison to those on smooth surfaces (*p* < 0.05, Figure 4B). Additionally, i‐PRF and EMD significantly promoted the migration of human osteoblasts at 48 h (Figure 4A,B, *p* < 0.05). The results showed that A‐PRF+ induced around 50% increase compared to the control group, whereas the application of i‐PRF and EMD resulted in another significant additional increase, around threefold compared to controls (group IV, *p* < 0.05). These findings were also dependent on Ti topography, whereas a rough surface positively influenced cell migration compared to smooth Ti surface (*p* < 0.05).

### 3.2 Gene Expression Analysis

**FIGURE 5:**
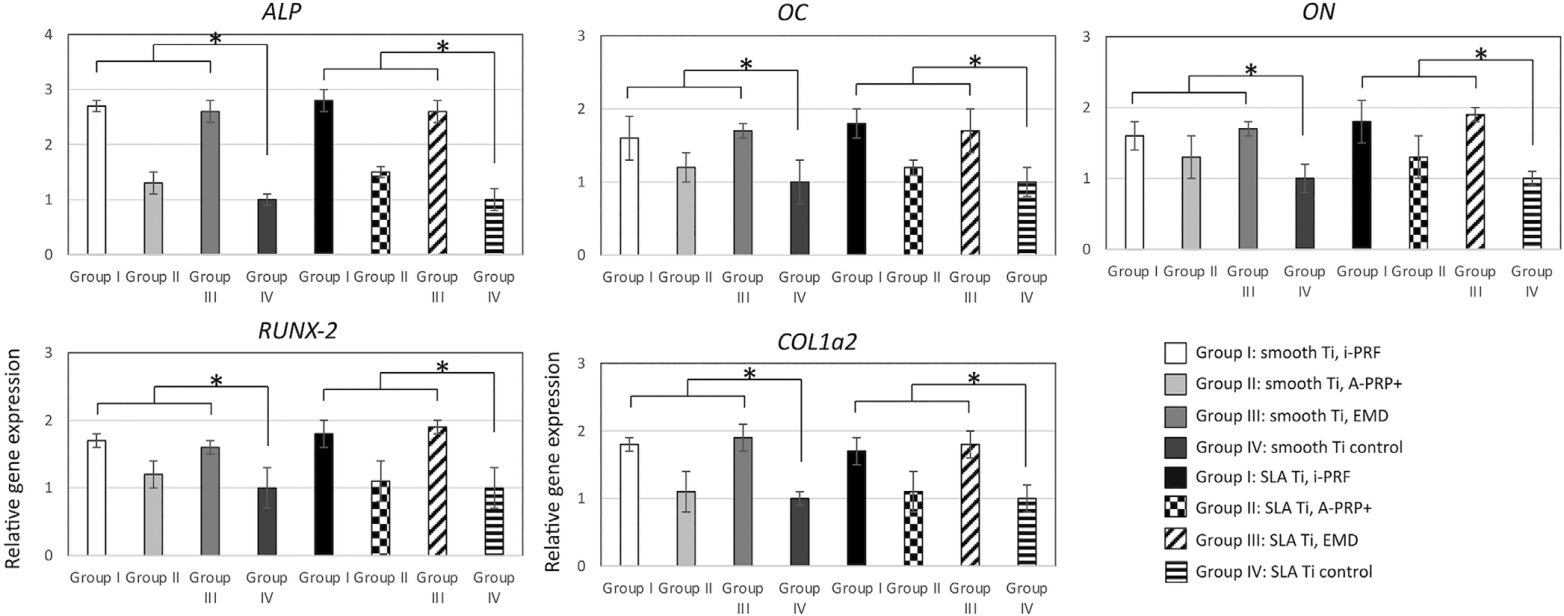
Gene expression analysis by RT‐PCR of human primary osteoblasts at day 7 cultured on smooth and rough Ti surfaces, with and without A‐PRF+, i‐PRF, and EMD for gene expression of *ALP* (alkaline phosphatase); *OC* (osteocalcin); *ON* (osteonectin), *RUNX‐2* (runt‐related transcription factor 2), *COL1a2* (collagen type I alpha 2). *A significant difference between control and experimental groups, *p* < 0.05.

The expression of mRNA levels of extracellular matrix‐related genes were evaluated by RT‐PCR at 7 days postseeding (Figure 5). The results showed that i‐PRF and EMD significantly increased *ALP, OC*, and *ON*, mRNA levels on all surfaces up to 2.8‐fold (*p* < 0.05) with no differences observed between Ti surface groups (smooth or rough Ti, *p* > 0.05). Thereafter, osteogenic differentiating markers including *RUNX‐2* and *COL1a2* expression were also evaluated by RT‐PCR. It was found that both i‐PRF and EMD provoked a significant upregulation in *RUNX‐2* and *COL1a2* mRNA levels when compared to their respective controls and A‐PRF+ (*p* < 0.05), with i‐PRF demonstrating significantly highest results with approximately 1.5‐fold at 7 days postseeding in smooth and rough surfaces.

## 4 Discussion

The present study aimed to compare the effects of two autologous platelet‐rich fibrin formulations (i‐PRF and A‐PRF+) and EMD on previously biofilm‐contaminated titanium discs. To the best of the authors knowledge, this study is the first to report the comparison between two different PRF protocols and EMD regarding their osteogenic potential and their effects on osteoblasts, respectively. The three biologic agents have been chosen for their underlying support in the literature, providing positive evidence for both hard and soft tissue augmentation for each of them. More specifically, liquid and solid PRF formulations have been tested against each other to find out which modality might be favorable for clinical application on implant surfaces.

Comparing the different PRF preparation protocols, no gold standard has been established so far. Regarding the RCF for liquid PRF preparation, two opposite opinions seem to persist: Whereas the group around Miron et al. advocated maximal centrifugation speed resulting in higher cellular and platelet concentration just above the BC layer (c‐PRF), Ghanaati et al. proposed the so‐called low‐speed centrifugation concept (LSCC) whereof the i‐PRF protocol that has been used in this study evolved [37]. LSCC for liquid fibrinogen matrices produces lower absolute plasma/fibrinogen layers with more homogenously distributed cells/platelets due to a decreased shift toward the BC layer. As a consequence, the same volume of a differently processed liquid PRF will result in lower (i‐PRF) or higher (c‐PRF) relative platelet and/or cell counts. Therefore, it would be interesting for further research to compare these two modalities against each other regarding identic or similar outcome measures as in the current study since biologically optimal concentrations of especially growth factors might not only correlate with bigger quantities. With regard to solid PRF preparation protocols, various parameters apart from RCF might have an impact on biological activity of the platelet concentrates. Some authors propagate advantages of horizontal centrifugation using so‐called “swing‐out” centrifuges, resulting in more homogenous cell and platelet distribution within the fibrin clot. Other researchers found no difference in PRF matrices produced either via fixed‐angle vs. horizontal centrifugation [38, 39]. Studies also have shown that horizontal centrifugation of PRF is more effective in accumulating platelets and leukocytes compared to the traditional fixed‐angle centrifuges used for producing solid PRF [38, 39]. Additionally, a novel technique was employed to obtain a concentrated form of i‐PRF from the BC layer directly above the red blood cell layer, which yielded a higher concentration of platelets and leukocytes. The researchers demonstrated 10‐fold capability to concentrate platelets and leukocytes by harvesting 0.3–0.5 mL of concentrated PRF (C‐PRF) from the BC following the L‐PRF protocol. They concluded that the C‐PRF protocols acquired huge platelet yields and could be further utilized for tissue regeneration [38]. Comparing liquid with solid PRF matrices, it has to be considered that in contrast to the homogenous distribution of cell and platelets in liquid matrices (regardless of the concentration), a solid PRF clot (irrespective of mechanical compression) will always exhibit an inhomogeneous gradient with most cells/platelets toward the BC layer. Using particulate solid matrices, for the clinician, it remains unclear whether one particle contains more or less biological activity. Nevertheless, the findings demonstrated that A‐PRF+, i‐PRF, and EMD provided a similar increase in osteoblast viability on smooth and rough Ti discs. These biologics also showed promise in promoting osteoblast adhesion and migration during the study. Notably, after 48 h, i‐PRF and EMD induced significantly higher osteoblastic cell migratory activity compared to A‐PRF+. These findings align with previous research which reported increase in osteoblast migration and proliferation with i‐PRF compared to conventional PRP [21, 33, 34, 35, 36, 40]. The study also noted that EMD promoted osteoblastic cell migration, consistent with previous research supporting its ability to stimulate periodontal tissue‐associated cells and promote periodontal and alveolar bone regeneration [35, 41]. Also, a noteworthy discovery was the enhanced migration of osteoblastic cells observed on rough‐treated titanium discs compared to smooth discs. This finding aligns with previous research suggesting that surface topography and roughness on titanium surfaces significantly influence cell responses and bone healing [42, 43]. Thus, biological modifications such as coating with i‐PRF on the implant surface could be a strategic approach to enhance osseointegration and reduce biofilm formation. In addition, the conditioned media prepared from i‐PRF and EMD appeared to promote the differentiation of preosteoblasts into mature osteoblasts by increased expression of osteoblastic differentiation markers after 7 days [17, 41, 44]. The findings of the study align with a previous study which also showed that i‐PRF exhibited maximum expression for genes encoding *OC* and *RUNX‐2*, key markers for osteoblastic activity (*RUNX‐2* and *COL1a2*) [21]. Similarly, the literature on EMD has demonstrated an increased expression of the *COL1a2* gene during osteoblast differentiation [22]. Furthermore, the study revealed an increased expression of *ALP, OC*, and *ON* genes on discs coated with i‐PRF and EMD, both on smooth‐ and rough‐treated surfaces. These findings are consistent with previous research [21, 34], showing that i‐PRF conditioned media increased *ALP* and *OC* mRNA levels compared to controls. Additionally, previous work demonstrated that the addition of EMD to osteoblasts resulted in the upregulation of genes related to bone mineralization and development [45]. The latter study also found a substantial increase in mRNA levels encoding bone sialoprotein, osteocalcin, and *ALP* in osteoblasts cultured on EMD‐coated surfaces [45]. Amelogenin also displayed a stimulatory effect on mRNA expression of osteopontin, osteonectin, and type I collagen, indicating its potential for bone regenerative therapies [34]. Indeed, EMD has been effectively utilized to regenerate intrabony defects around teeth affected by periodontal disease [46]. Various clinical studies have also shown that EMD, when used as an adjunct during implant placement or as a treatment for peri‐implantitis, contributes to improved implant survival rates over time [47, 48, 49].

The findings derived from our study underscore the favorable impact of i‐PRF, A‐PRF+, and EMD coating on the surface of titanium implants. A prominent advantage of this implant coating method is its simplicity and swiftness. In contrast to other surface alterations, i‐PRF coating is an entirely autogenous, biologically based, chairside procedure that promotes bone growth and may possess antibacterial properties. An appealing aspect of this approach is that it allows any conventional implant to be chairside modified by the operator, potentially enhancing both clinical and patient acceptance without significantly increasing the procedural costs. Moreover, this method could prove advantageous in cases where implant outcomes are expected to be compromised, such as in individuals with conditions like diabetes mellitus, osteoporosis, or at previously infected implant sites. One constraint of our research lies in its in vitro nature. Therefore, it is imperative to conduct additional long‐term clinical studies with a considerable sample size to substantiate the findings derived from this investigation. Furthermore, it is worth noting that the composition and effectiveness of platelet concentrates can vary significantly depending on factors such as gender, age, systemic health, smoking habits, ethnicity, and diet, as previously observed [50]. Clinicians should be mindful of the quality of devices used, such as ensuring they are registered or approved as medical devices by regulatory authorities, and be aware of the presence of potentially “hidden” chemical additives in PRF tubes. For instance, silica‐coated plastic tubes, which are becoming popular as alternatives to glass tubes, generate a distinct type of fibrin matrix with differences in platelet distribution and contamination by silica particles, which can adversely affect cell survival and proliferation [51].

Additionally, we acknowledge certain limitations in our current report related to in vitro cell analyses. While PRF is commonly employed in regenerative dentistry, in vitro models can only capture specific aspects of bone and wound regeneration, whereas in vivo models offer a more comprehensive perspective. Furthermore, present PRF research predominantly focuses on a limited range of cell types, overlooking the involvement of granulocytes, lymphocytes, and other cell varieties in bone regeneration and wound healing. It is essential to emphasize that the effects of the same stimuli can vary depending on the developmental stage of the target cells. Moreover, this study uses beta‐glycerophosphate and dexamethasone to induce osteogenic differentiation in vitro, mimicking aspects of osteogenesis in vivo. However, as an in vitro model, this cannot fully replicate the complexity of in vivo osteogenesis, which involves various cell types, mechanical loading, systemic hormones, local signaling molecules, and vascularization. While the model provides valuable insights into bone formation mechanisms, its results should be cautiously interpreted when applied to the more complex in vivo environment.

## 5 Conclusions

Based on the findings presented and within the limitations of this study, it can be concluded that coating of titanium discs with i‐PRF, A‐PRF+, and EMD causes positive increase in osteoblast viability, adhesion, migration, and proliferation response. This advantage, coupled with the fundamental benefits of autologous platelet concentrates, positions i‐PRF as a suitable agent for implant re‐osseointegration. However, considering the cellular activities of the tested PRF formulation and EMD, our research supports the notion that both i‐PRF and EMD are valuable bioactive materials that can be recommended to enhance tissue healing and improve bone formation and regenerative outcomes.

## Author Contributions

**Liza Lima Ramenzoni:** concept/design, Methodology, Validation, Data Analysis/Interpretation, Investigation, Data collection, Writing – Drafting article, Critical revision of the article, Statistics. **Jothi Varghese:** concept/Design, Methodology, Validation, Data Analysis/Interpretation, Investigation, Data collection, Writing – Drafting article, Critical revision of the article, Statistics, Approval of article, Project administration, Funding secured. **Patrick Roger Schmidlin:** concept/Design, Resources, Critical revision of the article, Approval of article, Project administration, Funding secured. **Shubhankar Mehrotra:** critical revision of the article, Resources, Project administration, Approval of article, Funding secured.

## Ethics Statement

The Ethics Committee of the Albert‐Ludwigs‐University Freiburg for research involving humans approved the study protocols (EK153‐15), and the study protocols followed World Medical Association Declaration of Helsinki guidelines.

## Consent

All authors have read and agreed to the published version of the manuscript.

## Conflicts of Interest

The authors declare no conflicts of interest.

## Data Availability

The data that support the findings of this study are available on request from the corresponding author. The data are not publicly available due to privacy or ethical restrictions.
